# Utility of clinical metagenomics in diagnosing malignancies in a cohort of patients with Epstein-Barr virus positivity

**DOI:** 10.3389/fcimb.2023.1211732

**Published:** 2023-08-22

**Authors:** Jieyu Song, Kun Zhu, Xiaojia Wang, Qingluan Yang, Shenglei Yu, Yi Zhang, Zhangfan Fu, Hongyu Wang, Yuanhan Zhao, Ke Lin, Guanmin Yuan, Jingxin Guo, Yingqi Shi, Chao Liu, Jingwen Ai, Haocheng Zhang, Wenhong Zhang

**Affiliations:** ^1^ Department of Infectious Diseases, Shanghai Key Laboratory of Infectious Diseases and Biosafety Emergency Response, National Medical Center for Infectious Diseases, Huashan Hospital, Shanghai Medical College, Fudan University, Shanghai, China; ^2^ Medical Department, Matridx Biotechnology Co., Ltd., Hangzhou, Zhejiang, China; ^3^ Huashan Institute of Microbes and Infections, Shanghai, China; ^4^ Shanghai Huashen Institute of Microbes and Infections, Shanghai, China

**Keywords:** infections, Epstein-Barr virus, high-throughput nucleotide sequencing, DNA copy number variations, differential diagnoses

## Abstract

**Backgrounds:**

Differentiation between benign and malignant diseases in EBV-positive patients poses a significant challenge due to the lack of efficient diagnostic tools. Metagenomic Next-Generation Sequencing (mNGS) is commonly used to identify pathogens of patients with fevers of unknown-origin (FUO). Recent studies have extended the application of Next-Generation Sequencing (NGS) in identifying tumors in body fluids and cerebrospinal fluids. In light of these, we conducted this study to develop and apply metagenomic methods to validate their role in identifying EBV-associated malignant disease.

**Methods:**

We enrolled 29 patients with positive EBV results in the cohort of FUO in the Department of Infectious Diseases of Huashan Hospital affiliated with Fudan University from 2018 to 2019. Upon enrollment, these patients were grouped for benign diseases, CAEBV, and malignant diseases according to their final diagnosis, and CNV analysis was retrospectively performed in 2022 using samples from 2018 to 2019.

**Results:**

Among the 29 patients. 16 of them were diagnosed with benign diseases, 3 patients were diagnosed with CAEBV and 10 patients were with malignant diseases. 29 blood samples from 29 patients were tested for mNGS. Among all 10 patients with malignant diagnosis, CNV analysis suggested neoplasms in 9 patients. Of all 19 patients with benign or CAEBV diagnosis, 2 patients showed abnormal CNV results. The sensitivity and specificity of CNV analysis for the identification for tumors were 90% and 89.5%, separately.

**Conclusions:**

The application of mNGS could assist in the identification of microbial infection and malignancies in EBV-related diseases. Our results demonstrate that CNV detection through mNGS is faster compared to conventional oncology tests. Moreover, the convenient collection of peripheral blood samples adds to the advantages of this approach.

## Introduction

Epstein-Barr virus (EBV) is a γ-herpesvirus with a double-stranded DNA genome that infects over 90% of the human population worldwide (de-The et al., 1975). It is mainly transmitted through saliva. Once inside the body, it invades the pharyngeal lymphatic tissue and B lymphocytes, leading to lifelong latent infections. EBV-related diseases often manifest as prolonged or unknown fever (FUO). The initial infection with EBV typically results in infectious mononucleosis, characterized by symptoms such as persistent fever, swollen lymph nodes, fatigue, malaise, etc (Dunmire et al., 2015). However, persistent EBV infection can have more serious consequences, including the development of Burkitt lymphoma, Hodgkin lymphoma, nasopharyngeal carcinoma, gastric carcinoma, and various malignancies in individuals with compromised immune systems due to either inherited or acquired immunodeficiency (Old et al., 1966; zur Hausen et al., 1970; Shibata et al., 1991; Hjalgrim et al., 2003; Thorley-Lawson and Gross, 2004). In addition to cancer, chronic active Epstein-Barr virus (CAEBV) is a progressive disorder associated with persistently high levels of EBV DNA in the blood and infiltration of organs by EBV-positive lymphocytes. Patients with CAEBV lack evidence of a known underlying immunodeficiency and are unable to control acute infection with the virus (Kimura, 2006; Kimura and Cohen, 2017). Moreover, they often exhibit poor response to antiviral therapy, interferon, intravenous immunoglobulin, and conventional chemotherapy, resulting in limited chances for improvement. In some cases, patients with CAEBV may develop EBV-positive B, T, or NK cell lymphomas (Okano, 2002). Given the severity of EBV-related diseases, it is crucial to promptly and accurately diagnose them to ensure appropriate treatment and care.

Methods used for the detection and monitoring of EBV include the heterophile antibody test, the EBV viral capsid antigen (VCA) IgG and IgM antibody test, EBV nuclear antigen (EBNA) antibodies, EBV-DNA qPCR and Epstein-Barr encoding region (EBER) *in situ* hybridization (Linderholm et al., 1994; Okano et al., 2005; Hurt and Tammaro, 2007; Gulley and Tang, 2008; De Paschale and Clerici, 2012). However, traditional microbiological tests that measure the pathogen load of EBV in blood cannot shed light on whether the patient’s symptoms are of an infectious or malignant etiology. Additionally, the pathogen load of EBV cannot be simply used to develop threshold values or patterns for medical intervention since it varies significantly between individuals and assay platforms (Dunmire et al., 2018; Wu et al., 2019), thus more focused clinical examinations are necessary. A pathological biopsy is used as the gold standard for malignancy diagnosis, but this operation might be higher-risk, time-consuming, laborious, and costly. It is call for a more convenient and rapid tests help with diagnosis in clinical practice.

Metagenomic Next-Generation Sequencing (mNGS) is a DNA sequencing-based approach for the detection of clinically relevant microorganisms. It has been commonly used in patients suspected of infectious diseases when a definitive etiological diagnosis is elusive (Chiu and Miller, 2019). Moreover, mNGS has clinical utilities that extend beyond pathogen detection (Leary et al., 2012). For instance, two recent studies have used mNGS to identify neoplasms from body fluids and cerebrospinal fluid with an overall sensitivity of 87% and specificity of 100% (Gu et al., 2021a; Gu et al., 2021b). This was achieved by analysis of chromosomal copy numbers of the host and detection of possible cancer-related copy number variations (CNVs). Although many other studies have been proved that the CNVs is the pathogenic factor of genetic diseases and could be used as a screening tool (Yang et al., 2013; Retterer et al., 2016; Trujillano et al., 2017; Zampaglione et al., 2020). Indeed, genomic instability is also one of the hallmarks of cancer cells (Shlien and Malkin, 2009), multiple studies have shown that CNV is an important component of genetic variation involved in the development and progression of tumors. Chromosomal CNVs have been recently used for early diagnosis of colorectal cancer, breast cancer, and hematologic malignancies (Kumaran et al., 2017; Xu et al., 2018; Lenaerts et al., 2019). For instance, pyothorax-associated lymphoma has been found to have the following characteristics: monoclonal pattern of EBV infection, complicated chromosomal abnormalities with numerous structural and numerical abnormalities, and occasional but distinct genome instability. These observations showed the potential utility of mNGS in diagnosing both microbial infections as well as cancer in patients who are positive for EBV in a single test (Takakuwa et al., 2003). In view of these, we developed and applied a metagenomic approach to validate its role in identifying EBV-related malignant diseases in this study.

## Materials and methods

### Study design and participants

**Figure 1:**
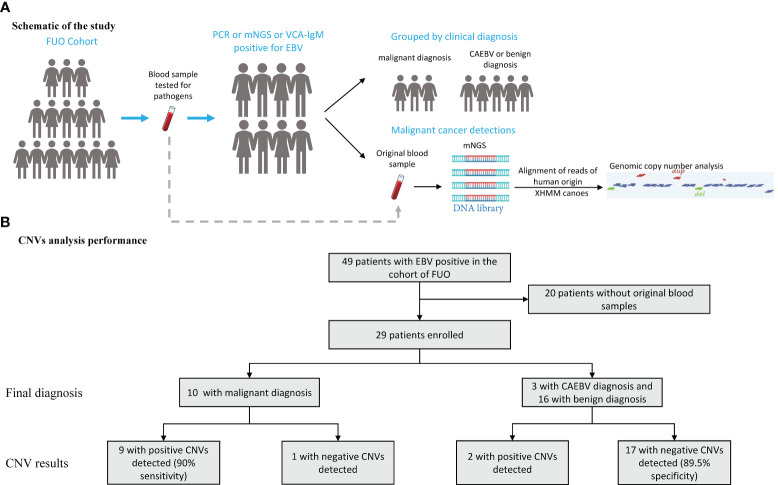
Schematic of the CNVs Test and Its Overall Performance. **(A)** An mNGS test was performed on blood samples assessed for aneuploidy and pathogens in patients with EBV positive in the cohort of FUO (fever of unknown origin). **(B)** 29 patients performed the mNGS test and the CNVs results manifested by groups. CNVs, copy number variations.

We enrolled the patients with positive EBV results in the cohort of FUO in the Department of Infectious Diseases of Huashan Hospital affiliated with Fudan University from 2017 to 2019. The peripheral blood samples were collected from the FUO patients on the first day of admission for the routine tests and pathogen detection examinations, which were stored simultaneously. The positive of EBV was ascertained by either (1) plasma or whole blood for EBV was positive by PCR or mNGS methods or (2) the EBV viral capsid antigen (VCA) IgM antibody test positive. The CNVs analysis was retrospectively performed in 2022 using original samples of the abovementioned EBV-positive patients from 2017 to 2019. And then these patients were classified into benign diseases, CAEBV, and malignant diseases based on their final diagnosis (**Figure 1A**). The final diagnosis was confirmed by 2 independent physicians with a follow-up after 5-7 years and the diagnostic process is described in the **Supplementary Information**. This study was approved by the Ethics Committee of Huashan Hospital (Approval number: KY2017-338).

### DNA extraction, library preparation and metagenomic sequencing

For DNA extraction, we used a kit from Matridx, Cat# MAR002 and followed standard operation procedures (SOPs) provided by the manufacturer. Peripheral blood samples were centrifuged at 16000g for 10 min and cell-free DNA (cfDNA) was extracted from plasma. DNA library was prepared by NGSmaster, a device that can automatically complete nucleic extraction, PCR-free library preparation (enzymatic fragmentation of genomic DNA, end repairing, terminal adenylation and adaptor ligation) and purification (Luan et al., 2021). Sequencing libraries were quantified by real-time PCR (KAPA) and pooled. Shotgun sequencing was carried out on Illumina Nextseq. Approximately 20 million of 50bp single end reads (dual barcode sequencing to minimize index hopping) were generated for each library. Bioinformatic analysis was conducted as described in a previous report (Shen et al., 2020). Briefly, sequences of human origin were filtered (GRCh38.p13) and the remaining reads were aligned to a reference database (NCBI nt, GenBank and in-house curated genomic database) to identify the microbial species and read count. For each sequencing run, a negative control (culture medium containing 10 (Hjalgrim et al., 2003) Jurkat cells/mL) was included.

### mNGS reporting criteria

The pathogens were reported if: 1) the sequencing data passed quality control filters (library concentration > 50 pM, Q20 > 85%, Q30 > 80%); 2) negative control (NC) in the same sequencing run does not contain the species or the RPM (sample)/RPM (NC) ≥ 5, which was determined according to previous studies as a cutoff for discriminating true-positives from background contaminations (Schlaberg et al., 2017; Wilson et al., 2019; Luan et al., 2021).

### CNVs analysis

DNA sequences were aligned to the human hg19 (GRCh37) reference genome. Guanine-cytosine (GC) content bias was corrected using LOESS regression. The standard deviation of the read fold change of each bin of data (bin size 100k) and normalized read counts was obtained. CNVs were called based on XHMM and Canoes (using reference data of normal chromosomal copy numbers) (Fromer et al., 2012; Backenroth et al., 2014). The sequencing depth was approximately 20M (20 million) reads to ensure that the coverage of the host chromosomes was comparable among specimens (>95% of sequencing reads were of human origin). The presence of large or multiple CNVs are strong indications of tumor cells since inherited CNVs are usually small (i.e. the largest inherited CNV is trisomy 21).We determined the possibility of neoplasms (presence of tumor cells) when large CNVs (> 10Mbp) were detected by the bioinformatic pipeline, which were unlikely to be caused by inherited disorders (Gu et al., 2021a). In addition, machine learning-based algorithm was applied to indicate the risk of tumors as previously described (Guo et al., 2021).

### Statistical analysis

The CNVs results were compared with the clinical final diagnosis for each patient. Sensitivity, specificity, efficiency, positive predictive value (PPV) and negative predicted value (NPV) were calculated to evaluate the ability of CNVs test in the performance of distinguishing the EBV-related diseases and malignant disease. In addition, we calculated Jouden’s index to conduct a comprehensive evaluation of the diagnostic indicator.

## Results

We enrolled 29 patients (median [interquartile range (IQR)] age, 40 [28-58] years; 8 (27.6%) women and 21 (72.4%) men), of whom 16 were diagnosed with benign diseases, 3 patients were diagnosed as CAEBV and 10 with malignant diseases (**Table 1**). We conducted mNGS analysis to identify chromosomal copy number variation (CNV) abnormalities. A total of 29 blood samples from 29 patients were examined.

**Table 1:** Patients’ characteristics and CNV results in the study.

Characteristics		
Patient Demographics		
**Age**	Median (IQR), y	40 (28,58)
** Sex**	Male, n (%)	21 (72.4)
	Female, n (%)	8 (27.6)
Final Diagnosis		
	Benign, n (%)	16 (55.2)
	CAEBV, n (%)	3 (10.3)
	Malignant, n (%)	10 (34.5)
CNV results		
	Positive, n (%)	11 (37.9)
	Negative, n (%)	18 (62.1)
**Lost to follow-up after discharged from hospital**	n (%)	5 (17.2)

The median (IQR) depth of sequencing was 11.9 (10.4-15.0) million reads. All 29 samples generate interpretable copy ratio plots (**Figure S1**). Of all the 29 patients, 10 patients were diagnosed with malignant diseases. And among all 10 patients with malignant diagnosis, there were 9 patients with positive CNV results (**Figure 1B**), including 5 cases lymphoma, 1 nasopharyngeal carcinoma, 1 gastric carcinoma, 1 liver cancer, and 1 case of polyneuropathy, organomegaly, endocrinopathy, M-protein, skin changes (POEMS) syndrome. Of all 19 patients with benign or CAEBV diagnosis, 2 patients showed abnormal CNV results. Thus, the sensitivity and specificity of CNVs analysis for the identification of tumors was 90% and 89.5%, separately (**Figure 1B**) and the efficiency is 89.7%. In general, the Jouden’s index of CNVs analysis is 0.76.

**Figure 2:**
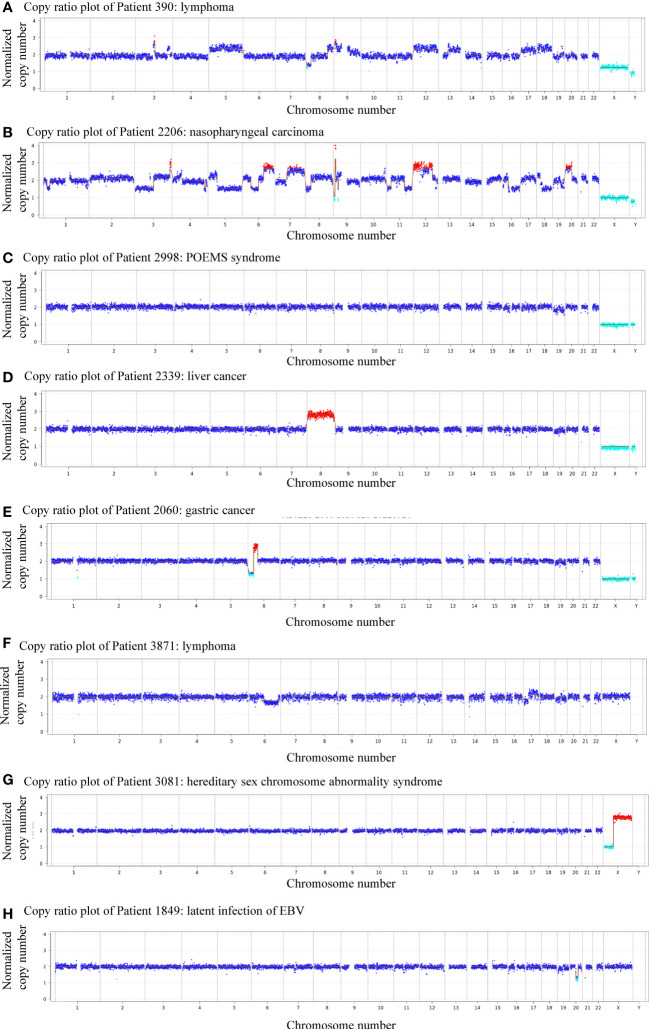
Plots showing patients with abnormal genomic copy numbers. **(A)** Copy ratio plot of Patient 390: lymphoma. **(B)** Copy ratio plot of Patient 2206: nasopharyngeal carcinoma. **(C)** Copy ratio plot of Patient 2998: POEMS syndrome. **(D)** Copy ratio plot of Patient 2339: liver cancer. **(E)** Copy ratio plots of Patient 2060: gastric cancer. **(F)** Copy ratio plot of Patient 3871: lymphoma. **(G)** Copy ratio plot of Patient 3081: superfemale syndrome. **(H)** Copy ratio plot of Patient 1849: latent infection of EBV.

Next-generation sequencing revealed the presence of more than 10 chromosomal copy number variations (CNVs) affecting over 10 million bases (10 M) of genomic regions in patients 390 and 2206, both diagnosed with malignant neoplasms. Specifically, patient 390 was diagnosed with lymphoma while patient 2206 was diagnosed with nasopharyngeal carcinoma (**Figures 2A, B**). Notably, four patients were thought to have CAEBV or benign diseases, but the CNV analysis suggested the possibility of cancer, which was confirmed by tissue biopsy or clinicians’ consensus in the follow-up evaluations. Among the four patients, one was diagnosed with POEMS syndrome, and CNV analysis indicated a deletion of *-19(mos)*, the CNV analysis revealed a duplication of *+8* in a patient with liver cancer, deletion of *6p22.2-p12.3(dup_23.5Mb)* and *6p25.3-p22.2(del[mos]_26.6Mb)* in a patient with gastric carcinoma, and a deletion of *6q11.1-q27(del[mos]_109.2Mb), 17p13.3-p11.1(del[mos]_22.3Mb)*, duplication of *17q11.1-q25.3(dup[mos]_55.9Mb)* and deletion of *19p13.3-p11(del[mos]_24.5Mb)* in a patient with lymphoma (**Figures 2C–F**). Among these chromosomal variations, abnormalities of chromosome 17 has been found to be related to lymphoma, loss and gain of chromosome 6 has frequently been reported in gastric carcinoma, and upregulated expression of chromosome 8 has been found in liver cancer (Jendiroba et al., 1995; Parada et al., 1998; Li et al., 2003; Kang et al., 2014; Li et al., 2022). These findings indicate the predictive and early diagnostic value of CNVs in malignant diseases.

Of all 11 patients that showed positive CNVs, two were finally diagnosed with benign diseases, indicating a positive predictive value (PPV) of 81.8%. Patient 3081 was a child diagnosed with superfemale syndrome and CNV indicated deletion of *Xp22.33-p11.22(del_49.9Mb)*, and duplication of *Xq11.1-q28(dup_93.3Mb)* (**Figure 2G**). On the other hand, patient 895 had a negative CNV result but was diagnosed with lymphoma, yielding a negative predicted value (NPV) of 94.4%. The follow-up of the other patient (#1849) was unavailable (**Figure 2H**).

## Discussion

Although chromosomal CNVs do not directly indicate cancer, they can provide valuable diagnostic clues for clinicians to order appropriate diagnostic tests. This can potentially save time, money and effort spent on discovering an infectious etiology, especially when microbiological tests are negative and empirical antibiotics had little effect. Differential diagnosis can be challenging and time-consuming, as different diseases may exhibit overlapping clinical manifestations. For instance, both benign and malignant diseases can result in elevated levels of serum inflammatory and tumor markers. Moreover, lymphoma can be misdiagnosed due to its ability to affect multiple organs and present with symptoms that resemble infection rather than cancer. Under such circumstances, CNVs detected by mNGS can lead to a more detailed clinical investigation of malignant diseases. Indeed, various studies have reported chromosomal abnormalities in cases of EBV-related malignancy, such as EBV-positive nodular sclerosis-type Hodgkin lymphoma, EBV-positive plasmablastic lymphoma (PL) carrying immunoglobulin (IG)/MYC rearrangements referred to a diagnostic hallmark of Burkitt lymphoma and EBV-positive PL with a higher mutation of JAK -STAT3 pathway genes (Taddesse-Heath et al., 2010; Valera et al., 2010; Castillo et al., 2015; Hayashida et al., 2017; Aukema et al., 2021; Ramis-Zaldivar et al., 2021). The EBV-related malignant diseases are serious conditions, but treatment is positive with equal harm caused by missed and misdiagnosis (Fugl and Andersen, 2019). The CNVs analysis with an efficiency of 89.7% could be helpful in daily clinical practice. Besides, Jouden’s index of this indicator is 0.76 which can be considered as an indicator of high diagnostic value.

Clinicians often encounter challenges when diagnosing and treating patients with fever of unknown origin, particularly when considering different potential causes such as microbial infections, malignancies, and collagen vascular diseases. These conditions often present with overlapping symptoms, making it difficult to determine the underlying cause. As a result, febrile cancer patients may receive empirical antibiotic treatment even in the absence of microbial infections due to delays in timely and efficient etiological diagnosis. Therefore, a diagnostic tool such as mNGS can be helpful to detect both pathogens and neoplasms and provide results in hours. One advantage of mNGS is that it does not require intact cells while pathological tests rely on the quantity and integrity of cells for proper analysis. In our study, the cell-free DNA (cfDNA) in plasma was processed for sequencing, which enables retrospective testing of stored specimens. By leveraging mNGS technology, clinicians can overcome the challenges of diagnosing FUO patients by obtaining comprehensive and rapid results that cover both infectious and malignant etiologies. This approach saves time, enables more targeted treatments, and offers the potential for improved patient outcomes.

The assay used in our study also has limitations. First, CNV analysis using mNGS data can only identify the gain or loss of genetic materials. Gene mutations (such as point mutations of oncogenes) cannot be detected due to limited sequencing depth (typically ~20 million reads for mNGS tests). In addition, unlike karyotyping, structural variations that do not involve numerical changes of gene copies cannot be detected, such as balanced translocation and inversion. As a result, tumors cannot be ruled out with a negative result. Second, experimental and quality control standards are currently lacking for mNGS, which are technically challenging to perform. It is recommended for febrile or immunocompromised patients when a chronic EBV infection has been diagnosed. Third, the CNVs detected by mNGS can only suggest the presence of tumor DNA but cannot pinpoint the location or whether the tumor is benign or malignant and therefore should be corroborated by additional diagnostic tests. Finally, the sample size in our study was limited, and clinical studies of a larger scale could yield more definitive evidence on the utility of mNGS in real-world settings.

In conclusion, the application of mNGS could assist in the identification of microbial infection and malignancies in EBV-related diseases. Our results demonstrate that CNV detection through mNGS is faster compared to conventional biopsy tests. Moreover, the convenient collection of peripheral blood samples adds to the advantages of this approach. On the other hand, it is important to note that this study is preliminary due to the limited sample size. Conducting prospective diagnostic trials on a larger scale would provide more conclusive evidence and enhance the validity of the findings.

## Data availability statement

The data presented in the study are deposited in the NGDC repository, accession number subCRA019136.

## Ethics statement

The studies involving humans were approved by Ethics Review Committee, Huashan Hospital, Fudan University, China. The studies were conducted in accordance with the local legislation and institutional requirements. Written informed consent for participation in this study was provided by the participants’ legal guardians/next of kin.

## Author contributions

HZ, JA, CL, and JS designed the study. XW and YS performed metagenomic sequencing experiments. JS, KZ, XW, CL, JA, and HZ analyzed data. JS, HZ, KZ, and XW wrote the manuscript. JA and CL revised the manuscript. All authors read and approved the final manuscript.

## References

[B1] Aukema S. M. Croci G. A. Bens S. Oehl-Huber K. Wagener R. Ott G.. (2021). Mantle cell lymphomas with concomitant MYC and CCND1 breakpoints are recurrently TdT positive and frequently show high-grade pathological and genetic features. Virchows Arch. 479, 133–145. doi: 10.1007/s00428-021-03022-8 33528622

[B2] Backenroth D. Homsy J. Murillo L. R. Glessner J. Lin E. Brueckner M.. (2014). CANOES: detecting rare copy number variants from whole exome sequencing data. Nucleic Acids Res. 42, e97. doi: 10.1093/nar/gku345 24771342 PMC4081054

[B3] Castillo J. J. Bibas M. MIranda R. N. (2015). The biology and treatment of plasmablastic lymphoma. Blood 125, 2323–2330. doi: 10.1182/blood-2014-10-567479 25636338

[B4] Chiu C. Y. Miller S. A. (2019). Clinical metagenomics. Nat. Rev. Genet. 20, 341–355. doi: 10.1038/s41576-019-0113-7 30918369 PMC6858796

[B5] De Paschale M. Clerici P. (2012). Serological diagnosis of Epstein-Barr virus infection: Problems and solutions. World J. Virol. 1, 31–43. doi: 10.5501/wjv.v1.i1.31 24175209 PMC3782265

[B6] de-The G. Day N. E. Geser A. Lavoué M. F. Ho J. H. Simons M. J.. (1975). Sero-epidemiology of the Epstein-Barr virus: preliminary analysis of an international study - a review. IARC Sci. Publ. 11 Pt 2 (1971), 3–16. 191375

[B7] Dunmire S. K. Hogquist K. A. Balfour H. H. (2015). Infectious mononucleosis. Curr. Top. Microbiol. Immunol. 390, 211–240. doi: 10.1007/978-3-319-22822-8_9 26424648 PMC4670567

[B8] Dunmire S. K. Verghese P. S. Balfour H. H. Jr. (2018). Primary Epstein-Barr virus infection. J. Clin. Virol. 102, 84–92. doi: 10.1016/j.jcv.2018.03.001 29525635

[B9] Fromer M. Moran J. L. Chambert K. Banks E. Bergen S. E. Ruderfer D. M.. (2012). Discovery and statistical genotyping of copy-number variation from whole-exome sequencing depth. Am. J. Hum. Genet. 91, 597–607. doi: 10.1016/j.ajhg.2012.08.005 23040492 PMC3484655

[B10] Fugl A. Andersen C. L. (2019). Epstein-Barr virus and its association with disease - a review of relevance to general practice. BMC Fam Pract. 20, 62. doi: 10.1186/s12875-019-0954-3 31088382 PMC6518816

[B11] Gu W. Talevich E. Hsu E. Qi Z. Urisman A. Federman S.. (2021a). Detection of cryptogenic Malignancies from metagenomic whole genome sequencing of body fluids. Genome Med. 13, 98. doi: 10.1186/s13073-021-00912-z 34074327 PMC8167833

[B12] Gu W. Rauschecker A. M. Hsu E. Zorn K. C. Sucu Y. Federman S.. (2021b). Detection of neoplasms by metagenomic next-generation sequencing of cerebrospinal fluid. JAMA Neurol. 78, 1355–1366. doi: 10.1001/jamaneurol.2021.3088 34515766 PMC8438621

[B13] Gulley M. L. Tang W. (2008). Laboratory assays for Epstein-Barr virus-related disease. J. Mol. Diagn. 10, 279–292. doi: 10.2353/jmoldx.2008.080023 18556771 PMC2438195

[B14] Guo Y. Li H. Chen H. Li Z. Ding W. Wang J.. (2021). Metagenomic next-generation sequencing to identify pathogens and cancer in lung biopsy tissue. EBioMedicine 73, 103639. doi: 10.1016/j.ebiom.2021.103639 34700283 PMC8554462

[B15] Hayashida M. Daibata M. Tagami E. Taguchi T. Maekawa F. Takeoka K.. (2017). Establishment and characterization of a novel Hodgkin lymphoma cell line, AM-HLH, carrying the Epstein-Barr virus genome integrated into the host chromosome. Hematol. Oncol. 35, 567–575. doi: 10.1002/hon.2369 27813134

[B16] Hjalgrim H. Askling J. Rostgaard K. Hamilton-Dutoit S. Frisch M. Zhang J. S.. (2003). Characteristics of Hodgkin's lymphoma after infectious mononucleosis. N Engl. J. Med. 349, 1324–1332. doi: 10.1056/NEJMoa023141 14523140

[B17] Hurt C. Tammaro D. (2007). Diagnostic evaluation of mononucleosis-like illnesses. Am. J. Med. 120, 911 e911–918. doi: 10.1016/j.amjmed.2006.12.011 17904463

[B18] Jendiroba D. Younes A. Katz R. Hill D. Cabanillas F. Andreeff M. (1995). Chromosome 17 numerical abnorMalities in 55 patients with non-Hodgkin's lymphoma: a fluorescence. Situ hybridization study. Leukemia 9, 1144–1146. 7630187

[B19] Kang M. Ding X. Xu M. Zhu H. Liu S. Wang M.. (2014). Genetic variation rs10484761 on 6p21.1 derived from a genome-wide association study is associated with gastric cancer survival in a Chinese population. Gene 536, 59–64. doi: 10.1016/j.gene.2013.11.087 24325909

[B20] Kimura H. (2006). Pathogenesis of chronic active Epstein-Barr virus infection: is this an infectious disease, lymphoproliferative disorder, or immunodeficiency? Rev. Med. Virol. 16, 251–261. doi: 10.1002/rmv.505 16791843

[B21] Kimura H. Cohen J. I. (2017). Chronic active epstein-barr virus disease. Front. Immunol. 8. doi: 10.3389/fimmu.2017.01867 PMC5770746 29375552

[B22] Kumaran M. Cass C. E. Graham K. Mackey J. R. Hubaux R. Lam W.. (2017). Germline copy number variations are associated with breast cancer risk and prognosis. Sci. Rep. 7, 14621. doi: 10.1038/s41598-017-14799-7 29116104 PMC5677082

[B23] Leary R. J. Sausen M. Kinde I. Papadopoulos N. Carpten J. D. Craig D.. (2012). Detection of chromosomal alterations in the circulation of cancer patients with whole-genome sequencing. Sci. Transl. Med. 4, 162ra154. doi: 10.1126/scitranslmed.3004742 PMC3641759 23197571

[B24] Lenaerts L. Vandenberghe P. Brison N. Che H. Neofytou M. Verheecke M.. (2019). Genomewide copy number alteration screening of circulating plasma DNA: potential for the detection of incipient tumors. Ann. Oncol. 30, 85–95. doi: 10.1093/annonc/mdy476 30371735

[B25] Li B. C. Chan W. Y. Li C. Y. Chow C. Ng E. K. Chung S. C. (2003). Allelic loss of chromosome 6q in gastric carcinoma. Diagn. Mol. Pathol. 12, 193–200. doi: 10.1097/00019606-200312000-00003 14639105

[B26] Li D. Pan J. Zhang Y. Li Y. Jin S. Zhong C.. (2022). C8orf76 modulates ferroptosis in liver cancer via transcriptionally up-regulating SLC7A11. Cancers (Basel) 14, 3410. doi: 10.3390/cancers14143410 35884471 PMC9316296

[B27] Linderholm M. BOman J. Juto P. Linde A. (1994). Comparative evaluation of nine kits for rapid diagnosis of infectious mononucleosis and Epstein-Barr virus-specific serology. J. Clin. Microbiol. 32, 259–261. doi: 10.1128/jcm.32.1.259-261.1994 8126196 PMC263013

[B28] Luan Y. Hu H. Liu C. Chen B. Liu X. Xu Y.. (2021). A proof-of-concept study of an automated solution for clinical metagenomic next-generation sequencing. J. Appl. Microbiol. 131, 1007–1016. doi: 10.1111/jam.15003 33440055

[B29] Okano M. (2002). Overview and problematic standpoints of severe chronic active Epstein-Barr virus infection syndrome. Crit. Rev. Oncol. Hematol. 44, 273–282. doi: 10.1016/s1040-8428(02)00118-x 12467967

[B30] Okano M. Kawa K. Kimura H. Yachie A. Wakiguchi H. Maeda A.. (2005). Proposed guidelines for diagnosing chronic active Epstein-Barr virus infection. Am. J. Hematol. 80, 64–69. doi: 10.1002/ajh.20398 16138335

[B31] Old L. J. Boyse E. A. Oettgen H. F. Harven E. D. Geering G. Williamson B.. (1966). Precipitating antibody in human serum to an antigen present in cultured burkitt's lymphoma cells. Proc. Natl. Acad. Sci. U.S.A. 56, 1699–1704. doi: 10.1073/pnas.56.6.1699 16591407 PMC220158

[B32] Parada L. A. Hallén M. Tranberg K. G. Hägerstrand I. Bondeson L. Mitelman F.. (1998). Frequent rearrangements of chromosomes 1, 7, and 8 in primary liver cancer. Genes Chromosomes Cancer 23, 26–35. doi: 10.1002/(sici)1098-2264(199809)23:1<26::aid-gcc5>3.3.co;2-g 9713994

[B33] Ramis-Zaldivar J. E. Gonzalez-Farre B. Nicolae A. Pack S. Clot G. Nadeu F.. (2021). MAPK and JAK-STAT pathways dysregulation in plasmablastic lymphoma. Haematologica 106, 2682–2693. doi: 10.3324/haematol.2020.271957 33951889 PMC8485662

[B34] Retterer K. Juusola J. Cho M. T. Vitazka P. Millan F. Gibellini F.. (2016). Clinical application of whole-exome sequencing across clinical indications. Genet. Med. 18, 696–704. doi: 10.1038/gim.2015.148 26633542

[B35] Schlaberg R. Chiu C. Y. Miller S. Procop G. W. Weinstock G. Professional Practice Committee and Committee on Laboratory Practices of the American Society for Microbiology. (2017). Validation of metagenomic next-generation sequencing tests for universal pathogen detection. Arch. Pathol. Lab. Med. 141, 776–786. doi: 10.5858/arpa.2016-0539-RA 28169558

[B36] Shen D. Song H. Wu X. Xu C. Su G.. (2020). Clinical assessment of the utility of metagenomic nextgeneration sequencing in pediatric patients of hematology department. Int. J. Lab. Hematol. 43(2), 244–249. doi: 10.1111/ijlh.13370 33099872

[B37] Shibata D. Tokunaga M. Uemura Y. Sato E. Tanaka S. Weiss L. M. (1991). Association of Epstein-Barr virus with undifferentiated gastric carcinomas with intense lymphoid infiltration. Lymphoepithelioma-like carcinoma. Am. J. Pathol. 139, 469–474. 1653517 PMC1886210

[B38] Shlien A. Malkin D. (2009). Copy number variations and cancer. Genome Med. 1, 62. doi: 10.1186/gm62 19566914 PMC2703871

[B39] Taddesse-Heath L. Meloni-Ehrig A. Scheerle J. Kelly J. C. Jaffe E. S. (2010). Plasmablastic lymphoma with MYC translocation: evidence for a common pathway in the generation of plasmablastic features. Mod Pathol. 23, 991–999. doi: 10.1038/modpathol.2010.72 20348882 PMC6344124

[B40] Takakuwa T. Luo W. J. Ham M. F. Mizuki M. Iuchi K. Aozasa K. (2003). Establishment and characterization of unique cell lines derived from pyothorax-associated lymphoma which develops in long-standing pyothorax and is strongly associated with Epstein-Barr virus infection. Cancer Sci. 94, 858–863. doi: 10.1111/j.1349-7006.2003.tb01367.x 14556658 PMC11160142

[B41] Thorley-Lawson D. A. Gross A. (2004). Persistence of the Epstein-Barr virus and the origins of associated lymphomas. N Engl. J. Med. 350, 1328–1337. doi: 10.1056/NEJMra032015 15044644

[B42] Trujillano D. Bertoli-Avella A. M. Kumar Kandaswamy K. Weiss M. E. Köster J. Marais A.. (2017). Clinical exome sequencing: results from 2819 samples reflecting 1000 families. Eur. J. Hum. Genet. 25, 176–182. doi: 10.1038/ejhg.2016.146 27848944 PMC5255946

[B43] Valera A. Balagué O. Colomo L. Martínez A. Delabie J. Taddesse-Heath L.. (2010). IG/MYC rearrangements are the main cytogenetic alteration in plasmablastic lymphomas. Am. J. Surg. Pathol. 34, 1686–1694. doi: 10.1097/PAS.0b013e3181f3e29f 20962620 PMC2982261

[B44] Wilson M. R. Sample H. A. Zorn K. C. Arevalo S. Yu G. Neuhaus J.. (2019). Clinical metagenomic sequencing for diagnosis of meningitis and encephalitis. N Engl. J. Med. 380, 2327–2340. doi: 10.1056/NEJMoa1803396 31189036 PMC6764751

[B45] Wu J. Lu A. D. Zhang L. P. Zuo Y. X. Jia Y. P. (2019). [Study of clinical outcome and prognosis in pediatric core binding factor-acute myeloid leukemia]. Zhonghua Xue Ye Xue Za Zhi 40, 52–57. doi: 10.3760/cma.j.issn.0253-2727.2019.01.010 30704229 PMC7351698

[B46] Xu J. F. Kang Q. Ma X. Y. Pan Y. M. Yang L. Jin P.. (2018). A novel method to detect early colorectal cancer based on chromosome copy number variation in plasma. Cell Physiol. Biochem. 45, 1444–1454. doi: 10.1159/000487571 29466793

[B47] Yang Y. Muzny D. M. Reid J. G. Bainbridge M. N. Willis A. Ward P. A.. (2013). Clinical whole-exome sequencing for the diagnosis of mendelian disorders. N Engl. J. Med. 369, 1502–1511. doi: 10.1056/NEJMoa1306555 24088041 PMC4211433

[B48] Zampaglione E. Kinde B. Place E. M. Navarro-Gomez D. Maher M. Jamshidi F.. (2020). Copy-number variation contributes 9% of pathogenicity in the inherited retinal degenerations. Genet. Med. 22, 1079–1087. doi: 10.1038/s41436-020-0759-8 32037395 PMC7272325

[B49] zur Hausen H. Schulte-Holthausen H. Klein G. Henle W. Henle G. Clifford P.. (1970). EBV DNA in biopsies of Burkitt tumours and anaplastic carcinomas of the nasopharynx. Nature 228, 1056–1058. doi: 10.1038/2281056a0 4320657

