# Supplementary material for: Utility of clinical metagenomics in diagnosing malignancies in a cohort of patients with Epstein-Barr virus positivity
Source: Front Cell Infect Microbiol. 2023 Aug 22;13:1211732. doi: 10.3389/fcimb.2023.1211732 (PMC10477599; doi:10.3389/fcimb.2023.1211732)
Supplement: Supplementary file 1 [file Table_1.docx]

**Supplementary Information**

**Methods**

A diagnosis of malignant diseases is eventually confirmed by either (1) a tissue diagnosis, or (2) a diagnostic consensus among hospital clinicians with an intention to treat. The diagnosis of CAEBV was ascertained by (1) persistent or recurrent IM symptoms for at least 3 to 6 months with markedly elevated titers to EBV lytic antigens (VCA IgG = 1:640 or EA IgG =1: 160), and (2) histologic evidence of major organ involvement is present such as interstitial pneumonia, hemophagocytosis, lymphadenitis, or persistent hepatitis, and (3) elevated EBV DNA, RNA or proteins are found by in situ hybridization or immunohistochemical staining of the affected tissues, and (4) high EBV viral loads in serum or PBMC by PCR.^1^ The patients received a clinical diagnosis of a pathogenic infection or autoimmune disease when they were confirmed with no known malignant cancer and CAEBV diagnosis.

**Table S1. The CNVs results of 29 Patients.**

| **Patient ID** | **CNVs** | | | **Discharge diagnosis** | **Final**  **diagnosis** |
| --- | --- | --- | --- | --- | --- |
|  | **CNVs result** | **Deletion** | **Duplication** |  |  |
| 390 | Positive | Yes | Yes | Malignant | Malignant |
| 510 | Positive | Yes | Yes | Malignant | Malignant |
| 895 | Negative | - | - | Malignant | - |
| 976 | Negative | - | - | CAEBV | CAEBV |
| 1127 | Negative | - | - | Bengin | Bengin |
| 1550 | Positive | Yes | Yes | Malignant | Malignant |
| 1849 | Positive | Yes | No | Bengin | Bengin |
| 1851 | Negative | - | - | CAEBV | CAEBV |
| 1861 | Negative | - | - | Bengin | Bengin |
| 2060 | Positive | Yes | Yes | Bengin | Malignant |
| 2206 | Positive | Yes | Yes | Malignant | Malignant |
| 2339 | Positive | No | Yes | Bengin | Malignant |
| 2909 | Negative | - | - | CAEBV | CAEBV |
| 2998 | Positive | Yes | No | Bengin | Malignant |
| 3081 | Positive | Yes | Yes | Bengin | Bengin |
| 3173 | Negative | - | - | Bengin | - |
| 3248 | Negative | - | - | Bengin | Bengin |
| 3269 | Negative | - | - | Bengin | - |
| 3426 | Negative | - | - | Bengin | Bengin |
| 3780 | Negative | - | - | Bengin | Bengin |
| 3871 | Positive | Yes | Yes | CAEBV | Malignant |
| 4233 | Positive | Yes | Yes | Malignant | Malignant |
| 5075 | Negative | - | - | Bengin | - |
| 5452 | Negative | - | - | Bengin | Bengin |
| 5620 | Negative | - | - | Bengin | Bengin |
| 5714 | Negative | - | - | Bengin | - |
| 6098 | Negative | - | - | Bengin | Bengin |
| 6100 | Negative | - | - | Bengin | Bengin |
| 6227 | Negative | - | - | Bengin | Bengin |

**Figure S1. Correlated sample and copy ratio plots of all 29 patients.**

Patient ID: 390

| Plasma | 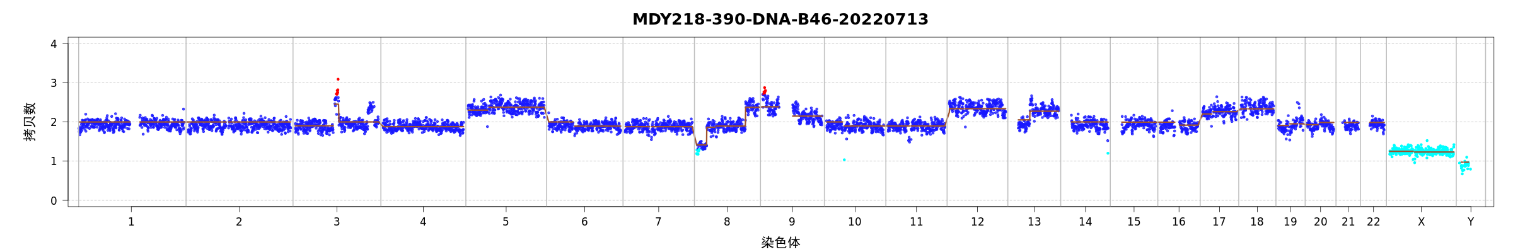  chromosomes  Normalized copy number |
| --- | --- |
| Total reads | 17918697 |
| CNVs abnormal | -11(mos)  +12(mos)  +17(mos)  +18(mos)  -4(mos)  +5(mos)  -7(mos)  +9(mos)  XY, +X(mos)  3p26.3-p11.1(del[mos]_90.5Mb)  3q11.1-q13.11(dup[mos]_9.7Mb)  6q11.1-q27(del[mos]_109.2Mb)  8p23.3-p21.2(del[mos]_23.9Mb)  8p21.2-p11.1(del[mos]_19.8Mb)  8q11.1-q23.3(del[mos]_69.1Mb)  8q23.3-q24.3(dup[mos]_30.4Mb)  10q11.21-q26.3(del[mos]_92.7Mb)  13q14.13-q34(dup[mos]_68.27Mb)  16q11.2-q24.3(del[mos]_43.9Mb) |

Patient ID: 510

| Plasma | 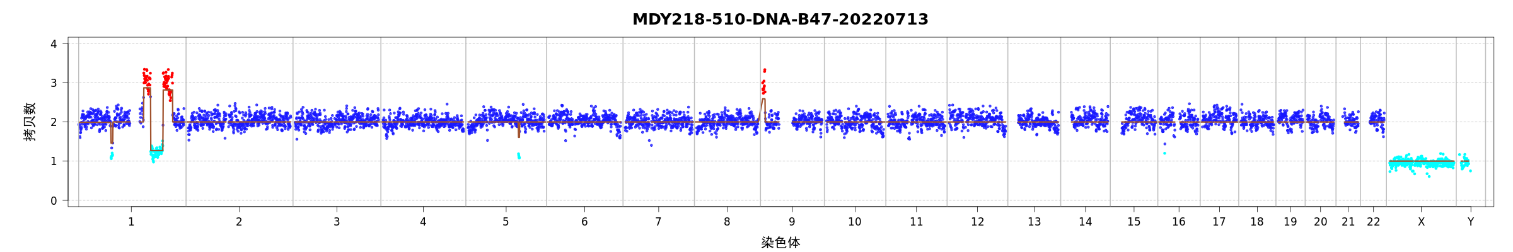  chromosomes  Normalized copy number |
| --- | --- |
| Total reads | 11438572 |
| CNVs abnormal | 1q21.3-q24.2(dup_16.5Mb)  1q32.1-q41(dup_22.7Mb)  1q24.2-q32.1(del[mos]_29.4Mb) |

Patient ID: 895

| Whole blood | 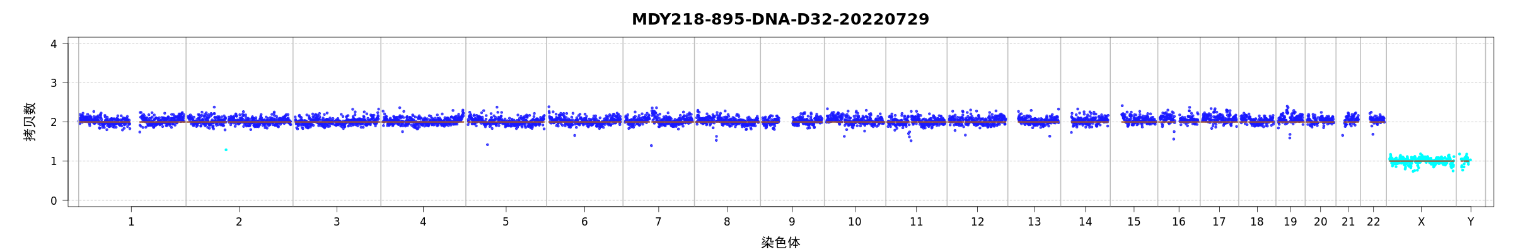  Normalized copy number  chromosomes |
| --- | --- |
| Total reads | 19998037 |

Patient ID: 976

| Whole blood | 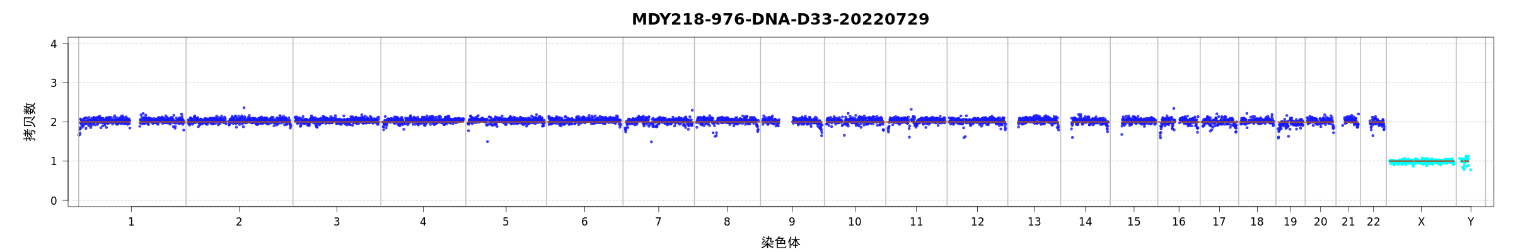  chromosomes  Normalized copy number |
| --- | --- |
| Total reads | 20308010 |

Patient ID: 1127

| Plasma | 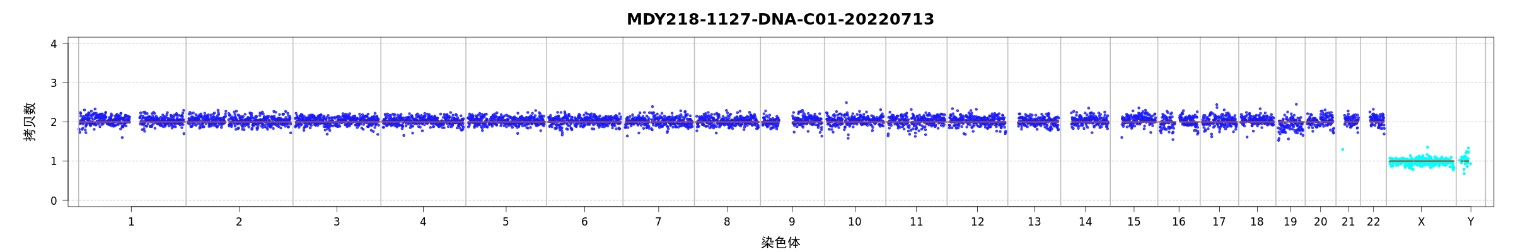  chromosomes  Normalized copy number |
| --- | --- |
| Total reads | 5353026 |

Patient ID: 1550

| Plasma | 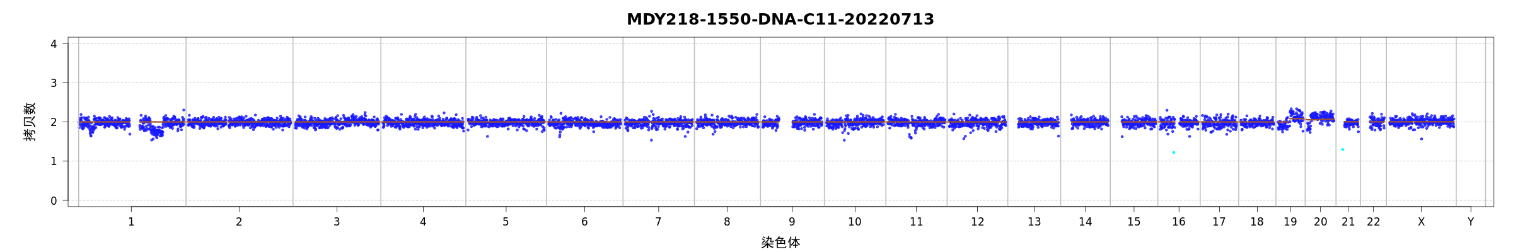  chromosomes  Normalized copy number |
| --- | --- |
| Total reads | 12590941 |
| CNVs abnormal | 20q11.1-q13.33(dup[mos]_33.6Mb)  1q24.2-1q32.1 del |

Patient ID: 1849

| Plasma | 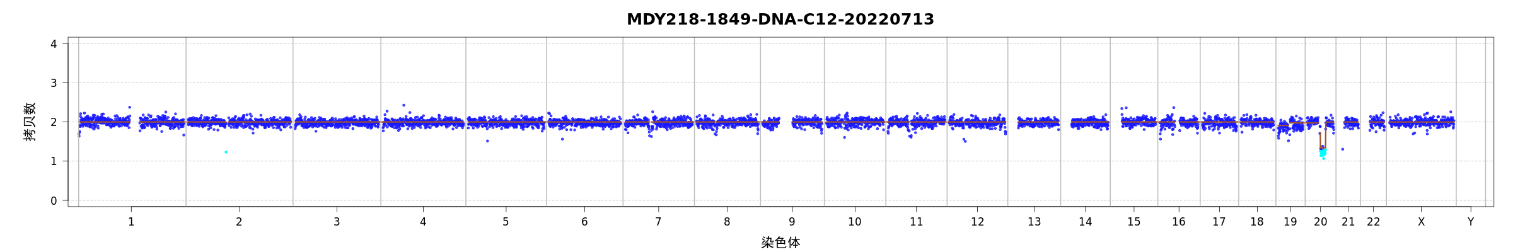  chromosomes  Normalized copy number |
| --- | --- |
| Total reads | 12224116 |
| CNVs abnormal | 20q11.21-q13.12(del[mos]_12.6Mb) |

Patient ID: 1851

| Whole blood | 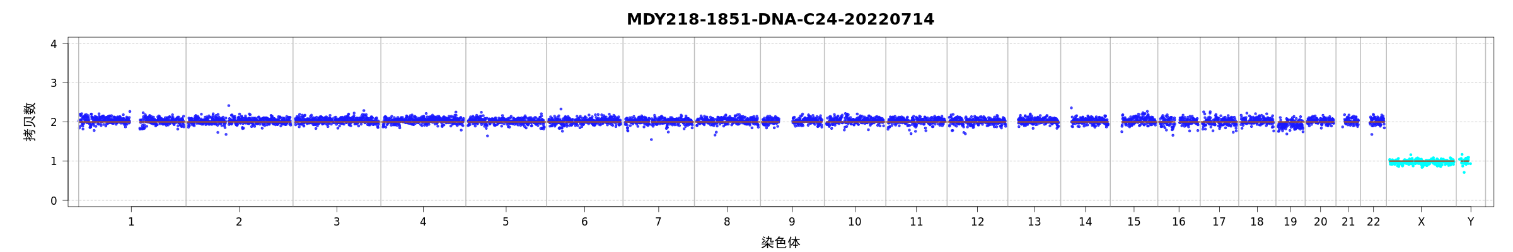  chromosomes  Normalized copy number |
| --- | --- |
| Total reads | 14788288 |

Patient ID: 1861

| Whole blood | 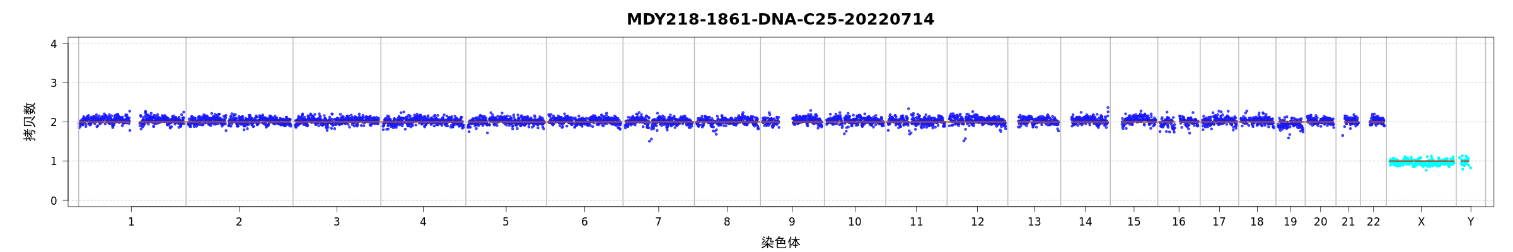  chromosomes  Normalized copy number |
| --- | --- |
| Total reads | 15538490 |

Patient ID: 2060

| Plasma | 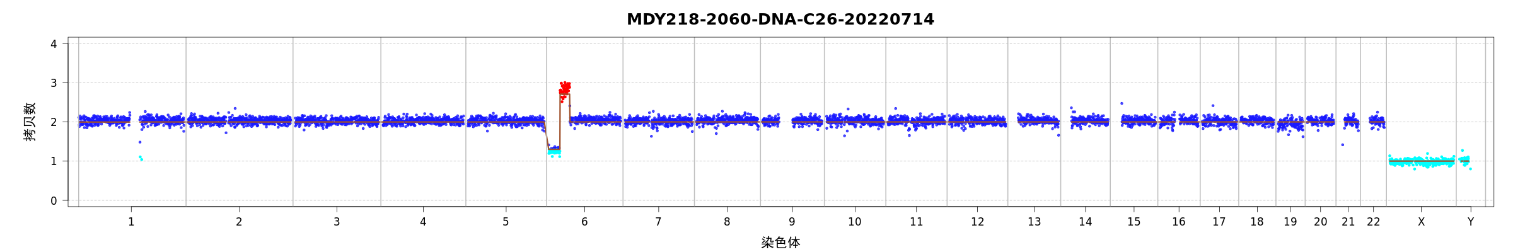  chromosomes  Normalized copy number |
| --- | --- |
| Total reads | 11057206 |
| CNVs abnormal | 6p22.2-p12.3(dup_23.5Mb)  6p25.3-p22.2(del[mos]_26.6Mb) |

Patient ID: 2206

| Plasma | 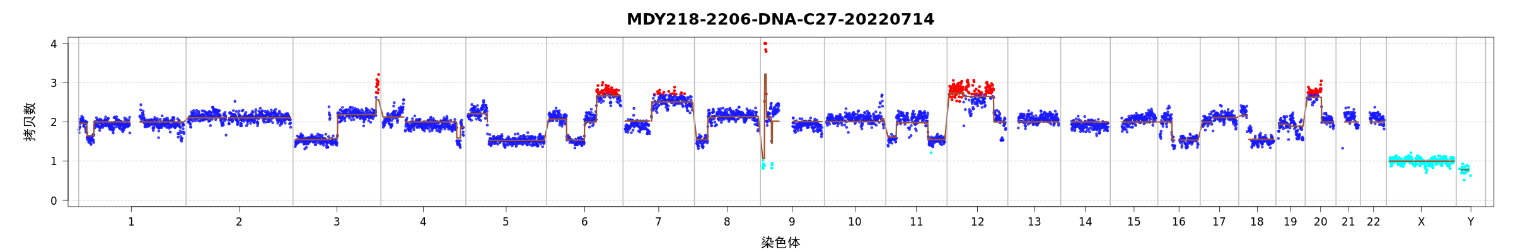  Normalized copy number  chromosomes |
| --- | --- |
| Total reads | 12922609 |
| CNVs abnormal | +2(mos)  XY, -Y(mos)  12p13.33-p11.1(dup_34.2Mb)  1p36.13-p34.3(del[mos]_16.6Mb)  3p26.3-p12.3(del[mos]_79.7Mb)  3q12.2-q28(dup[mos]_91.3Mb)  4p16.3-p11(dup[mos]_49.7Mb)  5p15.33-p12(dup[mos]_45.6Mb)  5q11.1-q35.3(del[mos]_131.4Mb)  6p21.1-p11.1(del[mos]_16.2Mb)  6q11.1-q14.2(del[mos]_23.1Mb)  6q21-q27(dup[mos]_57.7Mb)  7q11.21-q36.3(dup[mos]_96.4Mb)  8p23.3-p21.2(del[mos]_26.5Mb)  8q11.1-q24.3(dup[mos]_99.5Mb)  9p24.1-p21.3(dup[mos]_12.6Mb)  9p21.3-p11.2(dup[mos]_24.4Mb)  11p15.5-p15.1(del[mos]_19.7Mb)  11q21-q25(del[mos]_40.3Mb)  12q11-q23.3(dup[mos]_67.4Mb)  16q11.2-q24.3(del[mos]_43.9Mb)  17q11.1-q25.3(dup[mos]_55.9Mb)  18p11.32-p11.1(dup[mos]_15.5Mb)  18q11.1-q23(del[mos]_59.58Mb)  19q11-q13.43(del[mos]_31.4Mb)  20p13-p11.1(dup[mos]_26.4Mb) |

Patient ID: 2339

| Plasma | 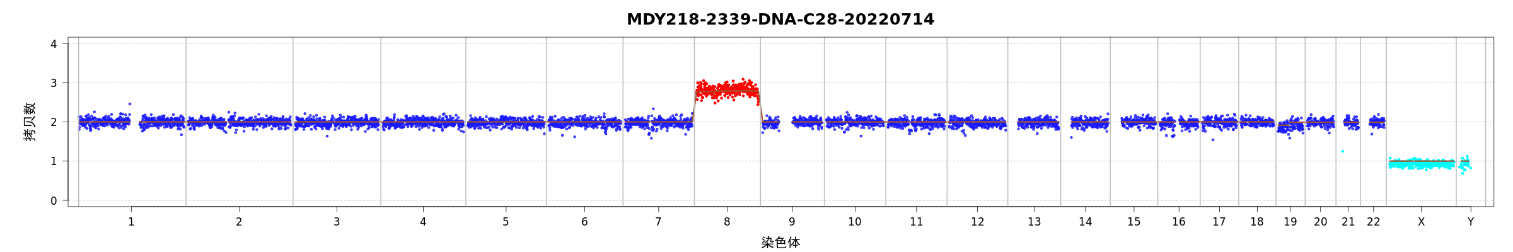  chromosomes  Normalized copy number |
| --- | --- |
| Total reads | 10412629 |
| CNVs abnormal | +8 |

Patient ID: 2909

| Plasma | 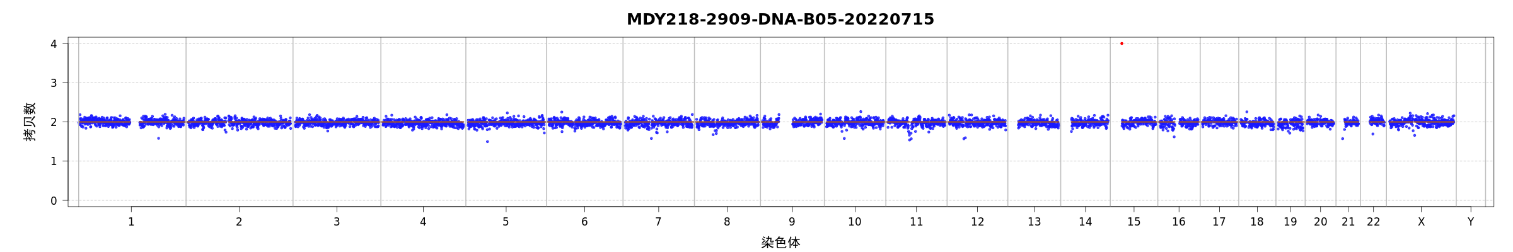  chromosomes  Normalized copy number |
| --- | --- |
| Total reads | 14348444 |

Patient ID: 2998

| Plasma | 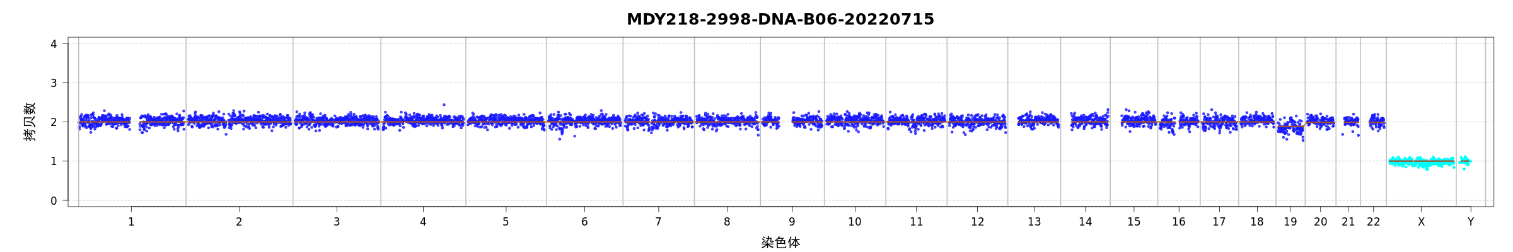  chromosomes  Normalized copy number |
| --- | --- |
| Total reads | 15016619 |
| CNVs abnormal | -19(mos) |

Patient ID: 3081

| Whole blood | 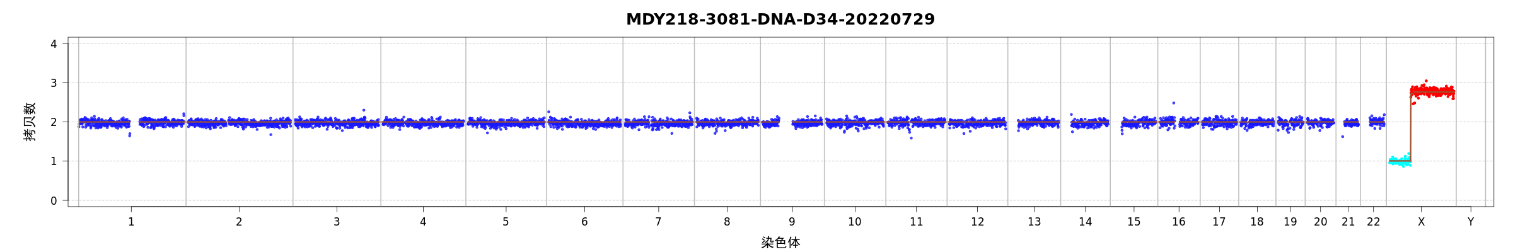  chromosomes  Normalized copy number |
| --- | --- |
| Total reads | 19454428 |
| CNVs abnormal | Xp22.33-p11.22(del_49.9Mb)  Xq11.1-q28(dup_93.3Mb) |

Patient ID: 3173

| Plasma | 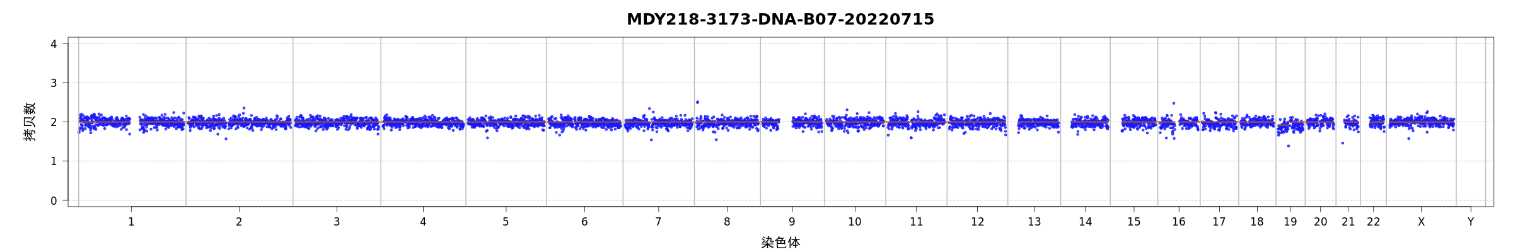  chromosomes  Normalized copy number |
| --- | --- |
| Total reads | 12019432 |

Patient ID: 3248

| Plasma | 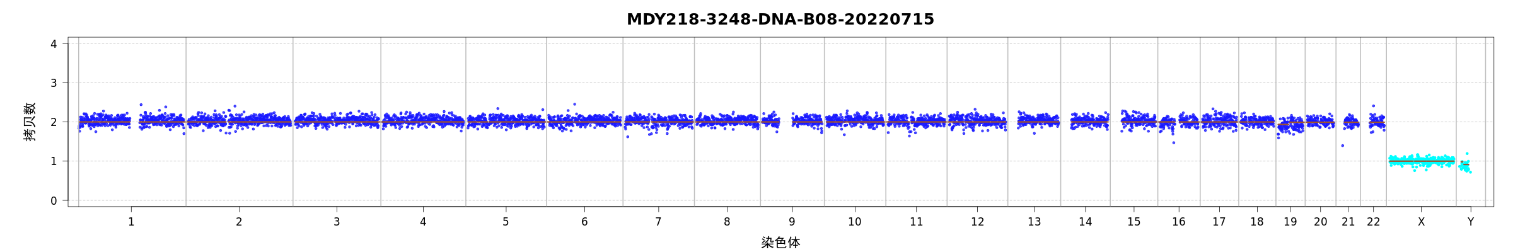  chromosomes  Normalized copy number |
| --- | --- |
| Total reads | 8468187 |

Patient ID: 3269

| Plasma | 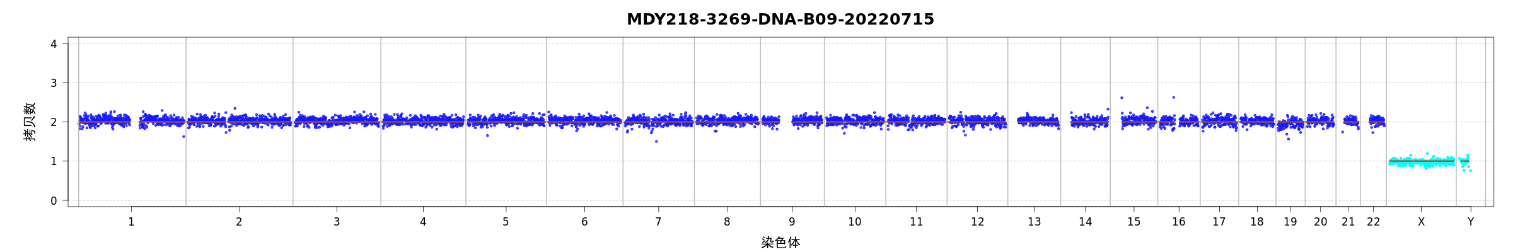  chromosomes  Normalized copy number |
| --- | --- |
| Total reads | 10496292 |

Patient ID: 3426

| Plasma | 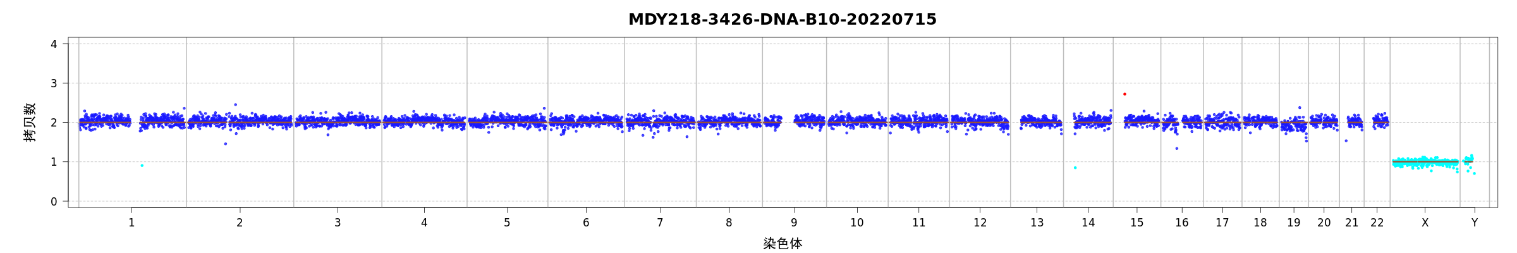  chromosomes  Normalized copy number |
| --- | --- |
| Total reads | 11866152 |

Patient ID: 3780

| Whole blood | 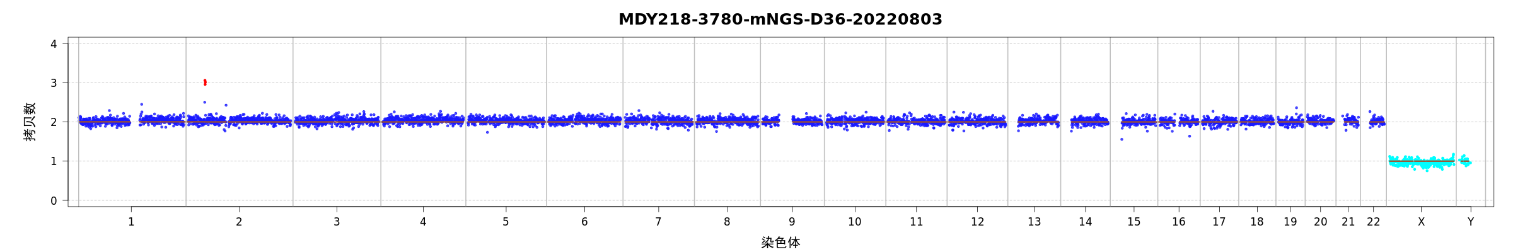  chromosomes  Normalized copy number |
| --- | --- |
| Total reads | 11700920 |

Patient ID: 3871

| Plasma | 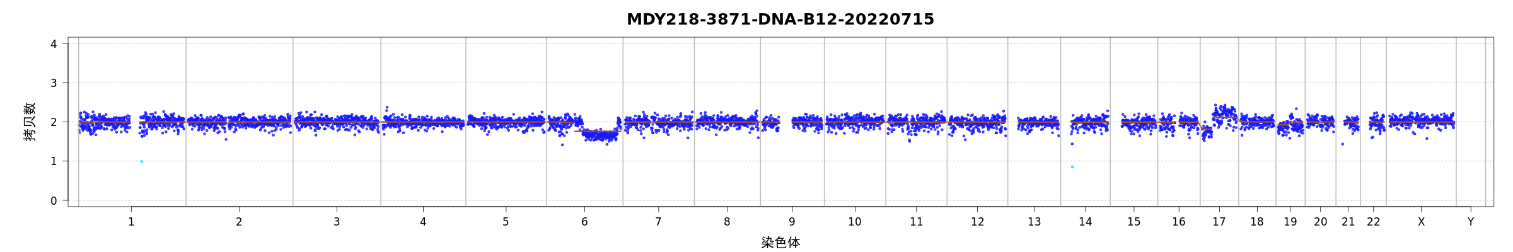  chromosomes  Normalized copy number |
| --- | --- |
| Total reads | 11524199 |
| CNVs abnormal | 6q11.1-q27(del[mos]_109.2Mb)  17p13.3-p11.1(del[mos]_22.3Mb)  17q11.1-q25.3(dup[mos]_55.9Mb)  19p13.3-p11(del[mos]_24.5Mb) |

Patient ID: 4233

| Plasma | 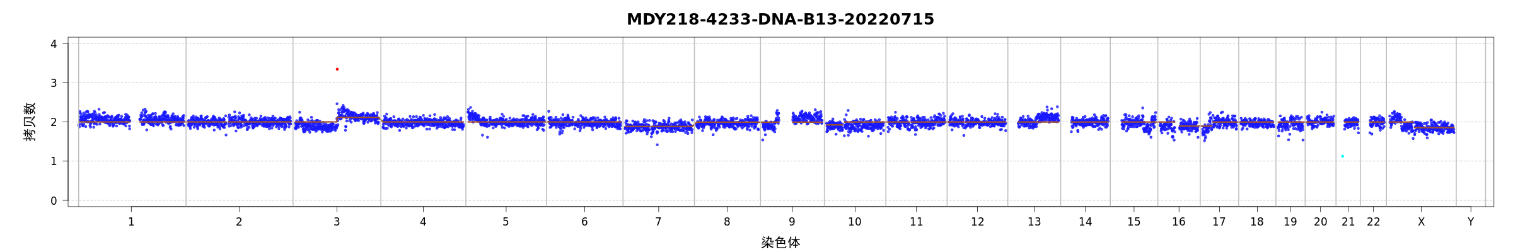  chromosomes  Normalized copy number |
| --- | --- |
| Total reads | 11701691 |
| CNVs abnormal | -7(mos)  3q12.1-q29(dup[mos]_97.9Mb)  10p15.3-p11.1(del[mos]_38.7Mb)  16q11.2-q24.3(del[mos]_43.9Mb)  17p13.3-p11.1(del[mos]_22.3Mb)  Xq11.1-q28(del[mos]_93.3Mb) |

Patient ID: 5075

| Whole blood | 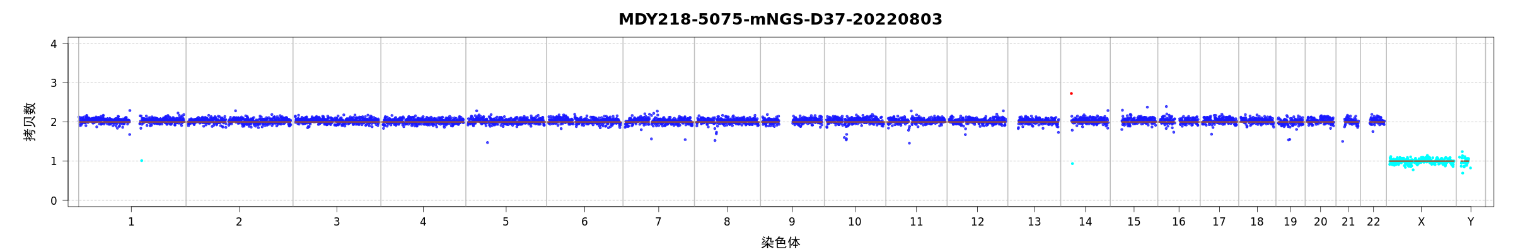  chromosomes  Normalized copy number |
| --- | --- |
| Total reads | 15902354 |

Patient ID: 5452

| Plasma | 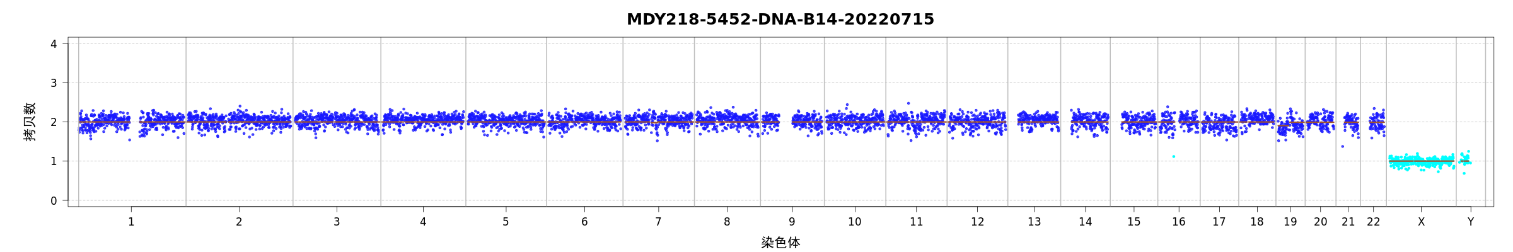  chromosomes  Normalized copy number |
| --- | --- |
| Total reads | 8644013 |

Patient ID: 5620

| Plasma | 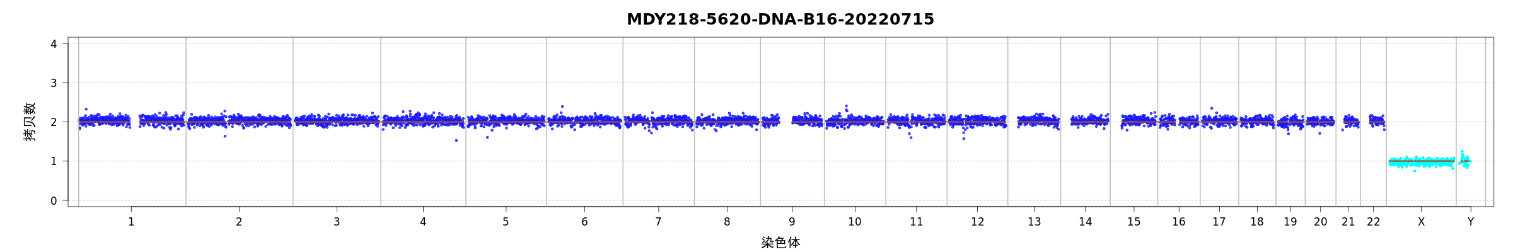  chromosomes  Normalized copy number |
| --- | --- |
| Total reads | 10101378 |

Patient ID: 5714

| Plasma | 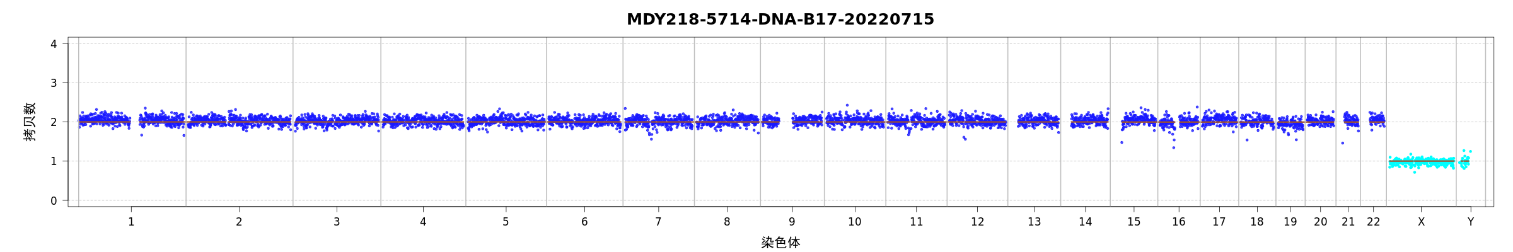  chromosomes  Normalized copy number |
| --- | --- |
| Total reads | 8489499 |

Patient ID: 6098

| Plasma | 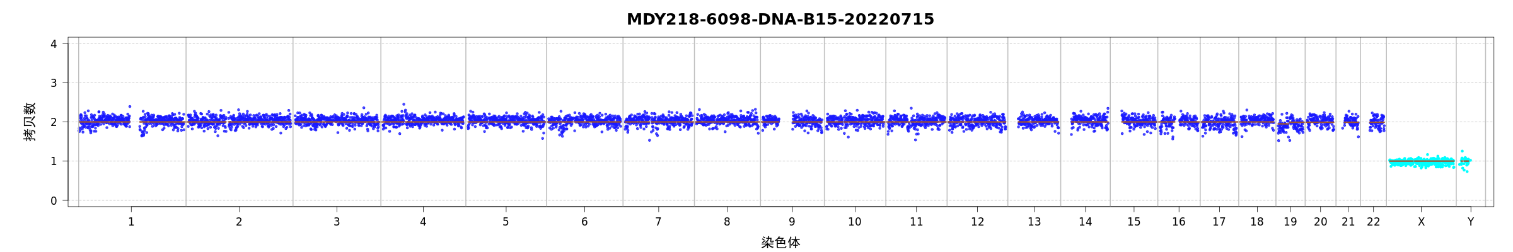  chromosomes  Normalized copy number |
| --- | --- |
| Total reads | 8783937 |

Patient ID: 6100

| Plasma | 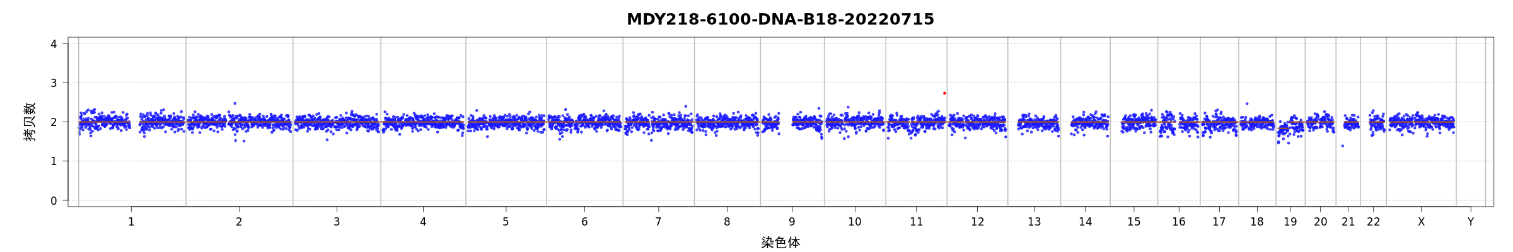  chromosomes  Normalized copy number |
| --- | --- |
| Total reads | 5720633 |

Patient ID: 6227

| Whole blood | 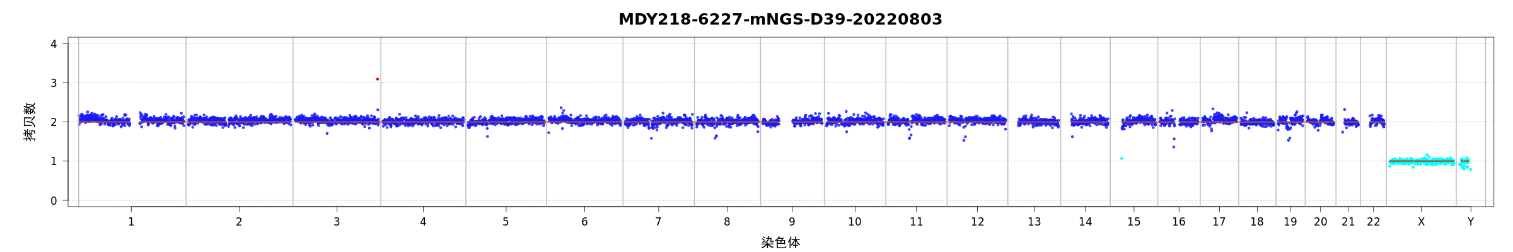  chromosomes  Normalized copy number |
| --- | --- |
| Total reads | 17732158 |

**reference**

1 Okano, M. *et al.* Proposed guidelines for diagnosing chronic active Epstein-Barr virus infection. *Am J Hematol* **80**, 64-69, doi:10.1002/ajh.20398 (2005).
